# How cultural capital, habitus and class influence the responses of older adults to the field of contemporary visual art^☆^

**DOI:** 10.1016/j.poetic.2013.07.001

**Published:** 2013-10

**Authors:** Andrew Newman, Anna Goulding, Christopher Whitehead

**Affiliations:** International Centre for Cultural and Heritage Studies, School of Arts and Cultures, University of Newcastle upon Tyne, Newcastle upon Tyne NE1 7RU, UK

**Keywords:** Older people, Contemporary visual art, Cultural capital, Habitus, Class

## Abstract

This article explores the responses of 38 older people to contemporary visual art through the results of a 28-month study entitled, *Contemporary Visual Art and Identity Construction: Wellbeing amongst Older People*. A framework for the analysis is provided by previous work on the consumption of art and by Bourdieu's constructs of cultural capital, habitus and field. Five groups of older people, with a range of different backgrounds, were taken to galleries and their responses were recorded, transcribed and analysed. It is concluded that participants’ responses are influenced by their cultural capital, habitus and class—which, in turn, are affected by their life course experiences. Those who could not recognise the field (e.g., did not view contemporary art as “art”) created their own meanings that they associated with the artworks. Evidence indicates that group dynamics and class mobility are likewise important. Participants also used the experience to respond to real or anticipated age-associated deficits.

## Highlights

• We explore the responses of 38 older people to contemporary visual art.
• We interpreted the results using Bourdieu's cultural capital, habitus and field.
• Those who could not recognise the field created their own meanings.
• Group dynamics and class mobility helped to determine responses.
• Participants also used the experience to respond to age associated deficits.

## 1 Introduction

This article explores how 38 older people experienced the field of contemporary visual art by analysing the results of a 28-month (May 2009–October 2011) study based in northeast England, UK. The study was funded by the New Dynamics of Ageing Programme^1^—which is an 8-year initiative designed to improve the lives of older people and is supported by five UK government research councils. The project involved recruiting five groups of older people from a variety of different backgrounds, including people who had a history of visiting art galleries and people who did not. The five groups ranged from the homogeneous (of a similar social class and age) to the heterogeneous (of a wider range in social class and age). Once the groups were selected, baseline data—for example, attitudes towards art and demographic information—were recorded, and then each group was taken to view different exhibitions at local galleries three times over the duration of the project. Groups had a guided tour, and their members were then asked (in focus groups) to reflect upon what they had seen—their responses were recorded, transcribed and analysed. As part of this larger project, the present article develops analysis presented by Newman (2013), which was used to interrogate assumptions that underpin cultural policy derived from the New Public Management (Dunleavy and Hood, 1994).^2^

In order to understand the data provided by the groups of older people, the exhibitions that they attended (and the galleries at which those exhibitions were held) are viewed as having positions within a Bourdieusian field (Bourdieu, 1984; Grenfell and Hardy, 2003, 2007; Reay, 2004; Swartz, 1997). The field positions of the galleries enable them to make specific epistemological propositions and claims with regard to “art”—for example, about the value of a particular artist's work or an art form.

Responses to contemporary visual art can be seen as being determined by respondents’ cultural capital (Bourdieu, 1997), habitus (Bourdieu, 1984; Reay, 2004) and social class (Silva, 2006), which may or may not have changed over their life course. For some people, navigating the field of contemporary visual art may be effortless and unconscious. However, for other people, it may represent fraught terrain because of the perception that contemporary art has elite cultural status while also being opaque in its meaning and requiring special cultural competencies among those who appreciate it (Whitehead, 2012). The above constructs (e.g., habitus), however, do not occur in a vacuum. For example, ageing, period and cohort effects (Alwin et al., 2006, explained below) are understood as impinging upon habitus. While period and cohort effects are not observable in qualitative studies such as this one, the impacts of life course experience and the social, psychological and physical effects of ageing are discernible.

The question that this article addresses is thus as follows: What role do cultural capital, habitus and class play in the responses of the older adults in the research project to the field of contemporary visual art—and are those constructs shaped by aspects of the life course (e.g., ageing)? This article is organised in the following way. Firstly, research on the consumption of culture is briefly explored, particularly that dealing with the visual arts. After this, two studies looking at the consumption of art by older people are described, including one that focuses upon barriers to their engagement. The theoretical model used is then presented, followed by a description of the data and methods, the analysis, and, finally, the concluding discussion.

## 2 The consumption of the visual arts

There is a long history of studies of participation in cultural activities, including participation in the visual arts. For example, Schuster (2007) identifies 20 different studies covering 35 countries that were mainly carried out for management or policy purposes, such as tracking progress towards achieving targets for increasing attendance at arts events in the UK. The lack of comparability in the ways the data were collected and how terms were defined means that generalised statements about the nature of participation across the datasets concerned are difficult to make and, hence, Schuster's paper focuses upon methodological issues. No conclusions are drawn about the influence of specific factors, such as social class, on participation.

However, such factors are considered by a quantitative study of social stratification and cultural consumption that considered the visual arts as part of the analysis, reported upon by Chan and Goldthorpe (2007). This used the 2001 Arts in England Survey carried out by the Social Survey Division of the UK Office for National Statistics on behalf of Arts Council England. They conclude that patterns of consumption in the visual arts are stratified mainly in terms of “status and education” (Chan and Goldthorpe, 2007, p. 184) and that the highly educated remain the main audience at art galleries.

According to Bennett et al. (2009), consumption of the visual arts generally has been neglected as an area of study. As part of the *Cultural Capital and Social Exclusion*^3^ project, which aimed to replicate and to update Bourdieu's (1984) work, the authors explored qualitative responses to J.M.W. Turner's *The Fighting Temeraire Tugged to Her Last Berth to be Broken Up* (1838) and David Hockney's *Paper Pools* (1980), as well as examining general visual arts consumption in England through interviews and focus groups and through a quantitative survey. They surmise that, in the visual arts, “disagreements about taste are found to be class-based, but other differences, particularly those of gender, age and ethnicity, intersect and at times change the inflections of class” (Bennett et al., 2009, p. 130). The ways that the influence of class is inflected by other differences is also seen in the work of Halle (1993). In his study, art in homes was examined in four areas of the New York region that provided a cross section of social classes and neighbourhoods. The taste for depopulated landscapes, for example, cut across the classes but the paintings chosen to represent that theme in homes in Manhattan (most advantaged) and Greenpoint (least advantaged) were influenced by respective class positions. Of importance to this article are the findings about the consumption of avant-garde or abstract art. Halle (1993, p. 122) finds that abstract art is “an elite taste, concentrated among sections of the middle and upper classes.” Yet, he notes, even amongst the most advantaged, a sizable minority disliked abstract art. A common view from all class groupings amongst those who disliked this art form was that it had no meaning and was, therefore, fraudulent. For those who liked it, its decorative qualities were important as well as its perceived capacity to provoke an imaginative response. Halle concludes that the meanings encoded by artists are not necessarily imposed or transmitted and that viewers often construct their own meanings in response to artworks.

Whereas the above works points to such things as class, Scherger (2009) demonstrates the importance of age as a variable influencing cultural consumption, doing so through an analysis of the UK Government Department for Culture, Media and Sport's *Taking Part* survey.^4^ She states that, “most social science studies of cultural activity focus on class, gender and ethnicity and at best treat age as a background variable that is rarely questioned further” (Scherger, 2009, p. 23). She notes that the “differences between different cultural practices can be traced back to underlying dimensions such as “the (questionable) hierarchy of practices, how far physical health and wellbeing are required and whether special abilities are necessary to practice the activity” (Scherger, 2009, p. 30). However, the lessons she draws have to be qualified by that fact that the cross-sectional nature of the data means that “the interplay of different factors in age differences in cultural participation cannot be demonstrated in detail” (Scherger, 2009, p. 28). A possible reason given for the identified declining participation amongst older people is that particular cohorts may have been socialised differently. For example, moves towards multiculturalism and greater inclusivity (DiMaggio and Mukhtar, 2004, p. 190) may preferentially influence “cohorts born after the Second World War in comparison to their parents.” Scherger (2009, p. 41) concludes that “the reasons why a 60-year-old person does not go to the cinema are different to the reasons that a 20-year-old does not go,” as the influence of class and age interact.

Using the results of the *Taking Part* survey, as well, Keaney and Oskala (2007) state that the 55–64 age group is highly engaged with the arts but participation declines noticeably after the age of 65. While it could be predicted that the factors that determine arts engagement for the whole population would equally apply to older people, certain demographic characteristics that predispose people to non-engagement are more prevalent amongst the older population. These characteristics are having a limiting disability or illness, being on a low income, living alone, having low levels of educational attainment, and being of lower socioeconomic status (Keaney and Oskala, 2007, p. 339). They conclude, “education appears to be one of the strongest predictors of arts engagement, with increasingly high rates of attendance and participation amongst older adults with higher educational qualifications” (Keaney and Oskala, 2007, p. 345).

The analysis presented in this article adds to existing work through its focus on older people and through its qualitative approach. Quantitative studies can give information about “who is attending, how people behave and how the development in participation has been,” but not about why people behave as they do (Bille, 2008, p. 114). It is also evident that some of the existing qualitative work has limited explanatory value. For example, in Bennett et al. (2009), respondents are classified as “confident amateurs,” “relaxed consumers” and “defensive individuals”—categories that would benefit from more unpacking.

## 3 Theoretical framework

The responses of the participants in the project have been explored using Bourdieu's (1984) view that practice is produced through the interaction of the constructs of field, cultural capital and habitus. This perspective has been chosen because it represents a way of explaining human motivations and behaviours within unequal social environments, allowing some of the theoretically important themes to be understood in the context of gallery visiting. This approach also helps to explain the class differences mentioned in the previous section.

Bourdieu (1983, p. 312) states that the artistic field is a:

Field of forces, but it is also a field of struggles tending to transform or conserve this field of forces. The network of objective relations between positions [within the field] subtends and orients the strategies that the occupants of the different positions implement in their struggles to defend or improve their positions.

Bourdieu (1984, p. 226) also writes that, in a given field, “the relationship of distinction is objectively described within it, and is reactivated, intentionally or not with each act of consumption.” Seen in this light, the field of contemporary visual art is inherently unequal—some people's opinions about the meaning and value of the art hold more sway than do others. That is, some opinions have more legitimacy.

Legitimacy in the field of contemporary visual art is defined by those who have dominant field positions, such as certain artists, curators and critics. While there may be debate about the relative merits of particular artists and approaches, there is overall agreement about what constitutes good and bad artistic practice—even if one feature of the field of contemporary art is its accommodation of challenging new approaches, so long as they can achieve consensual legitimacy through positive critical acclaim (see Johnson et al., 2006). However, this legitimacy is not fixed but evolves over time with new work becoming popular while other work becomes less so (Braden, 2009). The classification of art followed in this analysis is provided by Grenfell and Hardy (2003, 2007), as rear-guard, consecrated avant-garde, and avant-garde. They state of an artist:

His or her passage through the field of his or her generation may be fast or slow according to the degree of recognised legitimacy bestowed on him or her. And his or her generation itself may establish a consecrated position, or simply pass out of the current field, which contains the rear-guard tradition as well as successive generations of avant-garde defined in opposition to it and each other. (Grenfell and Hardy, 2003, p .27)

It is anticipated that the status given to particular artists or venues, and the extent to which they are recognised by participants, will influence the responses of the participants in this research.

How might they respond? Swartz (1997, p. 125) identifies three different field strategies:

Conservation strategies tend to be pursued by those who hold dominant field positions and enjoy seniority in the field. Strategies of succession are attempts to gain access to dominant positions in a field and are generally pursued by new entrants. Finally, strategies of subversion are pursued by those who expect to gain little from the dominant groups.

These strategies are more associated with elite actors within the field, but the framework can be of benefit in understanding other groups’ behaviour. Subversion, in this instance, might involve challenging how art is defined by the field of contemporary visual art if the viewer is unable to make sense of (i.e., decode) art with which they are confronted. However, a weak field position might mean that such a challenge would not succeed and would have no purchase in the field itself. It may, nevertheless, have purchase with social groupings with similar habitus, but a weak field position may still mean that the limited means and resources available for mounting such challenges (e.g., writing letters to newspapers, not visiting) would be unlikely to lend any power to influence the legitimacy of the field. Feelings of exclusion or inclusion amongst respondents, then, might be theorised as relating to feelings of competence or incompetence associated with the ability to decode or recognise the field.

The inequalities within fields result, in part, from differences in cultural capital that is used by actors to position themselves. Bourdieu (1997, p. 47) sees cultural capital as existing in three forms. The first is in an embodied state, “in the form of long-lasting dispositions of the mind and body” (Bourdieu, 1997, p. 47), the accumulation of which involves investment in the self and involves external wealth being “converted into an integral part of the person” (Bourdieu, 1997, p. 48). The second is an objectified form “referring to objects such as, books, works of art that require specialised cultural abilities to use” (Swartz, 1997, p. 76). The third is an institutionalised state that refers to educational qualifications. While, historically, cultural capital was understood as being in “opposition to popular culture, it now encompasses items from popular repertoires” (Prieur and Savage, 2011, p. 578), being understood as “symbolically valued cultural accoutrements and attitudes” (Grenfell and Hardy, 2007, p. 30).

The third construct used in this article is that of habitus. Swartz (1997, p. 103) states that habitus “generates perceptions, aspirations, and practices that correspond to the structuring properties of earlier socialisation.” Reay (2004) explains habitus as consisting of four related aspects. The first of these is embodiment, where the “body is in the social world and the social world is in the body” (Reay, 2004, p. 432). The second views habitus as being able to generate a wide range of possible actions, but while allowing “individual agency, it also predisposes individuals towards certain ways of behaving” (Reay, 2004, p. 433). The third aspect views habitus as a compilation of collective and individual trajectories. The fourth views it as a complex interplay between past and present. Habitus is viewed as being responsive to the present environment: “…current circumstances are not there just to be acted upon, but are internalised and become yet another layer to add to earlier socialisations” (Reay, 2004, p. 434). Habitus can be replicated by “encountering a field that reproduces its dispositions” or it can be transformed, “enabling conditions of living that can be very different to initial ones” (Reay, 2004, p. 435).

This article explores how older person's engagement or non-engagement with the field of art (particularly the avant-garde) is shaped by their stocks of cultural capital and their habitus being marked by the inequalities that are inherent within them. These inequalities help to explain how participants accept or reject avant-garde art and the field strategies of conservation, succession or subversion that they sometimes adopt when faced with such art.

## 4 Data and methodology

A qualitative approach was adopted for this study, as it was more likely to gauge subtle shifts in affect over the life of the project (Denscombe, 2003, p. 167). Five groups of participants were recruited from the categories identified by Keaney and Oskala (2007) as being vulnerable to exclusion from the arts. These were: limiting disability or illness; low income; living alone; low levels of educational attainment; and lower socioeconomic status. However, we felt that to provide a point of comparison, it was also necessary to recruit some who did not fall into these categories. Contacts were provided by local organisations that were subsequently invited to become members of the project's management group. These organisations consisted of the BALTIC Centre for Contemporary Art; Gateshead Older People's Assembly; Equal Arts (an older person's arts agency for northeast England); the Northern Gallery for Contemporary Art, Sunderland; and the local branch of Age UK, a national UK charity promoting the rights of older people.

Some of the recruited groups of older people were pre-existing, while other groups came together specifically for this research, although their members already belonged to a common organisation. For example, the sheltered accommodation group members lived in the same place and knew each other, but they had not come together for activities before they volunteered to take part in the study. In the recruitment process, an appointment was made and the project introduced. The opportunity was then given to volunteer and details of those who agreed to take part were recorded. Ethnically, all participants were White British (Dobbs et al., 2006).^5^ Contacts were made with local black and minority ethnic groups, but none of those contacted wished to take part. It was decided not to recruit those suffering from dementia and ethical approval was applied for and received from Newcastle University, Newcastle upon Tyne, UK, on that basis. This was because the research team lacked the specialist skills needed to work with those with this condition.^6^

Data were collected at seven points during the project for each group. For the baseline data, one-to-one interviews were offered (as opposed to focus groups), and this offer was taken by the sheltered accommodation group. In total, 38 older people were recruited across the five groups with numbers of between 6 and 9 in each group, which allowed discussion to take place addressing a range of topics, including their educational histories, general perceptions about ageing, and attitudes towards contemporary visual art.

Each group visited three exhibitions over the lifetime of the project, the final one being chosen by the respondents. A description of the venues and shows attended is provided in Appendix A of the online supplement. The groups were taken to the venue by minibus or taxi, given lunch and then given a guided tour around the show by a curator or education officer (apart from Belsay Hall, Castle and Gardens, Northumberland—where a tour was not available). Focus groups were then used to record responses to the experience of the visits (see Wilkinson, 2004). Two members of the research team were present during the focus groups, with one being a moderator and the other observing and making notes. The stimuli for the discussion were the exhibition and gallery within which it was displayed. The moderator initiated the discussion, and then the group members discussed the exhibition and venue amongst themselves, responding to comments that others in the group made. Occasionally, the moderator would steer the conversation back to the exhibition and ensured that all were able to make a contribution. Participants responded to what they had seen and heard in the guided tour without a structure being imposed by the moderator. Occasionally, conversations between participants in the gallery were recorded.

The criteria used to choose the shows and venues were that they represented different positions in the field of contemporary visual art—being mainly avant-garde, but including some consecrated avant-garde and rear-guard works (see Grenfell and Hardy, 2003, 2007). It was also important to be able to visit the venues and return participants to their homes easily to prevent tiredness. Private spaces in venues were made available to act as a base, for discussions, as well as for refreshments.

The focus groups were of between 30 and 120 minutes duration and were recorded and transcribed. In total, there were 56 transcripts, which were then coded using Nvivo 9.^7^ The coding was informed by close reading of the transcripts by the research team, by previous work on the consumption of the arts generally and the consumption of the arts by older people in particular, and by the theoretical framework adopted. Inferences about respondents’ engagement were derived from reactions and strategies they were seen to employ during the visits.

The concepts introduced in the section on theory are used for the purposes of the analysis in the following way. The cultural capital of the participants is understood in terms of education—the institutionalised form mentioned by Bourdieu (1997). The significance of this, in the older population, is noted by Scherger (2009, p. 35), who states from her study of the UK's *Taking Part* survey (mentioned above) that, “two thirds of the respondents over 75 years old do not have any educational qualifications.” Secondly, how respondents interpret the art they saw is seen in terms of their habitus—as that is what shapes perceptions and dispositions (Swartz, 1997). Both habitus and cultural capital are implicated in class, with the latter defined by Silva (2008, p. 268) as “the positions in a hierarchical social order occupied by individuals in social space, according to which aesthetic engagements will vary.” The classification of social class in this article is based upon occupations as provided by “The National Statistics Socio-economic Classification” (NS-SEC).^8^ This consists of 8 groupings that are used to position the respondents as follows:

1. Higher managerial and professional occupations.
2. Lower managerial, administrative and professional occupations.
3. Intermediate occupations.
4. Small employers and own account workers.
5. Lower supervisory and technical occupations.
6. Semi-routine occupations.
7. Routine occupations.
8. Never worked or long-term unemployed.

Age, cohort and period effects, as described by Alwin et al. (2006), contribute to the habitus of participants. These authors state that the effects of historical time can be defined in terms of cohort effects, which are described as “the stable differences among birth cohorts as a result of the historical circumstances of their development” (Alwin et al., 2006, p. 22). Period effects can be identified “when an entire social group is affected by historical events, such as war, an economic depression, or social movement” (Alwin et al., 2006, p. 21). However, there are difficulties in using this model empirically. Idler (2006, p. 286) observes that “although age, cohort and period effects are conceptually distinct they are analytically inseparable (because any two determine the third) and more importantly they often appear in complex interactions.” Because of these difficulties, and the fact that cohort and period effects are normally associated with large-scale quantitative studies, no attempts are made to observe them directly within the qualitative analysis presented in this article. However, it is possible to consider the effects of certain life experiences that respondents might have had and how that influences the interpretations they make in response to the artworks. As their ages range from 61 to 90, differences in interpretation caused by this are expected. As ageing effects are viewed as the physical, psychosocial and social effects of ageing (Scherger, 2009), some evidence of these influencing responses is expected to be seen in individuals, and this is discussed in Section 5.5.

### 4.1 Groups recruited to the project

#### 4.1.1 Sheltered accommodation group, Gateshead, Tyne and Wear

This group consisted of 7 women with an age range of 62–90 who live in sheltered accommodation in Gateshead, Tyne and Wear. They found it difficult to live independently, and they had lived locally before taking up residence. All of the group, apart from two, were over 68; of the younger ones, a 64-year-old suffered from learning difficulties and a 62-year-old was deaf and had recently been widowed (and, subsequent to the research, she moved out of the sheltered accommodation unit). All apart from the 62-year-old (who had been a nurse) left school aged 14 or 15 and went into employment immediately working in occupations such as cook; factory worker; punch card operator; and sales person (NS-SEC classes 5, 6 and 7). They were strongly marked by their class origins and some class mobility was evident, with a 68-year-old obtaining a position as a salesperson for a local construction company. This group visited the BALTIC Centre for Contemporary Art to see *Parrworld* and *A Needle Women* (12 November 2009), the Shipley Art Gallery to see *Knitted Lives* (9 March 2010), and the Northern Gallery for Contemporary Art to see *Systematic* (15 June 2010).

#### 4.1.2 Writers’ group, Sunderland, Tyne and Wear

This group, which dated from 1986, consisted of 6 women who ranged from 64 to 87 years-old, 5 of whom were over 72. One member had “O” and “A” levels,^9^ while the others left school without qualifications at age 14 or 15. Three returned to educational courses, with one obtaining a degree at age 62. Occupations mentioned included: cook; cleaner; shop worker; nurse; social worker; probation officer; housewife; secretary; and factory worker. The occupations of this group would have originally placed them in classes 5, 6 and 7 of the NS-SEC. However, class mobility has meant that, for some, this changed over their life course. This group identified themselves proudly as working class. They visited the Northern Gallery for Contemporary Art to see *Rank* (17 June 2009), the BALTIC Centre for Contemporary Art to see *Parrworld* and *A Needle Women* (26 November 2009), and the Great North Museum: Hancock (24 March 2010).

#### 4.1.3 Group recruited from an advocacy organisation for older people, Gateshead, Tyne and Wear

This group consisted of 9 individuals, 6 females and 3 males, who ranged from 63 to 83 years old. This was a mixed group in terms of both age and class. Three were aged 63–64, while 6 were aged between 79 and 83. Occupations included: cook; civil servant; teacher; cabaret singer; private industry worker; dental nurse; shop manageress; and university researcher (younger NS-SEC groups 1 and 2 and older groups 2 and 3). Educational qualifications obtained ranged from none to a PhD. They visited the BALTIC Centre for Contemporary Art to see *Parrworld* and *A Needle Women* (26 November 2009), the Northern Gallery for Contemporary Art to see the show by *Semiconductor* (10 March 2010), and Belsay Hall, Castle and Gardens to see *Extraordinary Measures* (12 May 2010).

#### 4.1.4 Group recruited from a daytime film club for the over 60s in Newcastle upon Tyne, Tyne and Wear (henceforth called the “film group”)

This group contained 3 females and 4 males. They ranged in age from 61 to 65 years-old, with all but one being placed in NS-SEC classes 1 and 2. Previous occupations, included: primary school teacher; chartered engineer; social worker; and occupational psychologist; and one had a lower supervisory and technical (class 5) job at a local brewery. All, apart from the former brewery worker, were educated to degree level, with the primary school teacher returning to education and qualifying as a teacher after leaving school at 16. They visited the Northern Gallery for Contemporary Art to see the show by *Semiconductor* (23 March 2010), the BALTIC Centre for Contemporary Art to see a show by Jenny Holzer (13 May 2010), and a show by Anselm Kiefer (14 October 2010).

#### 4.1.5 Men's group recruited from a “live at home” scheme, Gateshead, Tyne and Wear

This group consisted of 9 men who ranged in age from 62 to 88 years old (the 62-year-old was disabled and the ages for the others ranged from 72 to 88 years). Previous occupations mentioned included: company director; maintenance electrician; clerk; painter and decorator; and maintenance fitter for a coalmine. Apart from a 73-year-old who had become a company director (despite having a similar background to the others), they were classed as NS-SEC groups 5, 6, and 7. The 62-year-old member of the group left school with “O” levels, while the others left at 14 or 15 without educational qualifications. Apart from the 73-year-old, they remained in their original occupation. However, some of their children and grandchildren demonstrated class mobility, taking middle class occupations such as teacher and gaining places at prestigious universities. They visited the Northern Gallery for Contemporary Art to see the show by *Semiconductor* (28 April 2010), the BALTIC Centre for Contemporary Art to see a show by Cornelia Parker (8 September 2010), and the Hatton Gallery to see *Hugh Stoneman: Master Printer—The Art Fund Archive* (3 November 2010).

It is not claimed that the above groups are representative of the older population, but they do allow the impact of cultural capital, habitus and class on the consumption of contemporary visual art to be explored.

## 5 Analysis

Differences in responses could be identified between broad groupings of working class older participants aged 68–90 years (comprising the sheltered accommodation and “live at home” scheme groups) and middle class younger participants aged 61–65 years (comprising the film group). However, class differences were blurred both in the writers’ group and the group recruited from the advocacy organisation, which had a more complex profile, being mixed in terms of class and life course trajectory. These latter groups are considered in separate sections below, as will a consideration of the general impact of ageing.

### 5.1 Working class older participants 68–90 years: “live at home” scheme and sheltered accommodation groups

While both these groups have individual younger members, the analysis here focuses on the older ones. These older participants remained strongly marked by their original socio-economic position with most—except for the 73-year-old who became a company director—continuing with their original occupations over their careers. Apart from this company director, the others had occupations classed as groups 5, 6 and 7 (NS-SEC) and had limited educational opportunities—with most leaving school at 14 or 15, which is a generational effect for this group.^10^

#### 5.1.1 Definitions of art

Within the habitus of participants are embedded ways of classifying and understanding art. When those embedded ways did not allow them to make sense of the contemporary art that was offered to them, then their obvious retort was to classify what they saw in the exhibitions as not art. This approach was typical amongst the working class older respondents.

After visiting the Northern Gallery for Contemporary Art to see *Heliocentric*, the 73-year-old member of the “live at home” scheme stated:

Art to me is when you take a hammer and chisel to a big slab of marble and make these wonderful things that you see round the world and also when you have a paintbrush in your hand and you see all these paintings of landscapes, birds, horses, cattle and things like that. That is art.

This demonstrates a form of recognition and engagement with the field of art more broadly, the rules of which—as understood by this participant—are not transferable to the field of contemporary visual art. The respondent's statement also reflects the common tendency—including among these working class respondents—to locate the value of artworks in their mimetic qualities (in line with mimetic or imitation theory of art). However, as Bourdieu (1993, p. 217) states:

There is no perception that does not involve an unconscious code and that minimum, and apparently immediate, comprehension, accessible to the simplest observers and enabling them to recognise a house or tree, still presupposes partial (unconscious) agreement between the artist and beholder concerning categories that define the representation of the real that a historic society hold to be realistic.

Bourdieu's point is that even valuing artworks according to their mimetic qualities involves a process of decoding; arguably, this process of decoding is not fundamentally different from the decoding that takes place when engaging with more complex artworks, wherein physical/visual mimesis is not paramount (e.g., conceptual art). But, nevertheless, it is evident that some individuals’ field positions, such as that flowing from their class, do not enable or encourage them to extend their decoding processes to engage constructively with more avant-garde art.

A member of the “live at home” scheme—who was a 73-year-old widower who had worked in shipyards and steelworks in Jarrow, Tyne and Wear—stated in response to works in the exhibition *Heliocentric* (which focus on the sun) at the Northern Gallery for Contemporary Art, “It was interesting to see the close ups of the sun, but I couldn’t associate it with art somehow. I just don’t understand what it was supposed to be.” This kind of answer is explained by Bourdieu (1993, p. 217), who states that “since the information presented by the works of art exceeds the deciphering capabilities of the beholder, he perceives them as devoid of signification—or, to be more precise, of structuration and organisation.”

An 83-year-old participant from the “live at home” scheme attributed this “not art” classification to age (rather than class), stating, “I think when you are younger, like in our day, art was a different thing, and you are used to what you were brought up with.” However, as abstract art dates at least to the end of the 19th century, it was present when he was younger. It is possible, instead, that unequal circulations of representations of contemporary art mean that an individual might not have been exposed to it. These respondents demonstrate a liking for forms of art that were representational and demonstrated craft skill, a definition identified in a previous study of older person's engagement with art (Newman et al., 2012).

#### 5.1.2 Decoding of artworks/interpretations of art viewed

When asked to respond to particular art pieces and galleries, those in the working class older group adopted different strategies—depending on the resources that they could bring to bear when being asked to reflect on the contemporary art works that they deemed “not art.” As an expression of participants’ habitus, the codes used by this grouping for everyday perception were applied to make sense of the art works. This included ways of understanding art in general—but also involved making links to aspects of participants’ personal or class histories, being closely associated with their generation, which allowed them to make sense of what they were looking at but had, in many cases, little relationship to the field of contemporary art in art historical terms. This was apparently carried out without difficulty and was typical of these respondents.

Of course, the ability of the respondents to make sense of the artworks using everyday codes was dependent on whether relevant themes could be identified. Consider, for example, the response of an 82-year-old female member of the sheltered accommodation group. When encountering the artwork, *Mountain Rescue Helicopter Gunship* (1992, Dinah Prentice), which had been inspired by the artist's experience as a child in wartime Germany, the older woman stated:

I was bombed out three times during the war. If you haven’t seen that, you can’t imagine that, I can. The church was completely wiped out and a school friend of mine was being taught the organ. He was killed.

Respondents in this grouping used the themes they identified in the artworks to make personal links with important events in their and their community's history. This woman from the sheltered accommodation was making such a link between the artwork and a traumatic event in her childhood. In this sense, an act of interpretation did take place, but the object of interpretation was the respondents’ life experience rather than the artwork, which acted as a catalyst for the interpretive process. Among this study's participants, this response can only have been produced by someone of this age who had experienced the effects of World War II at first hand. However, as Bottero (2010, p. 13) states, even if others had shared these experiences, it does not guarantee similar responses to the artwork because “how to behave must be interpreted and operationalised in each given circumstance, and because the coordination of habitus depends upon group dynamics,” suggesting that the social circumstances of the visit may also play a part in enabling her response.

In drawing on such everyday codes, the significance of class, gender and generation was shown during the visit of the sheltered accommodation group to the *Knitted Lives* exhibition at the Shipley Art Gallery. The pieces were made in conjunction with two artists, and they reflected the domestic lives of older women in northeast England. The Shipley Art Gallery has a specialist collection in the sub-field of contemporary craft, and this—along with the resemblance of some of the pieces to mixed-media sculptural installations, which are not uncommon in the field of contemporary art—allowed the gallery to make a value claim about knitting that both subverts and depends on traditional hierarchies of art. The pieces constructed included a (knitted) midwife's bike, a cake and make-up. While the participants from the sheltered accommodation did not have knowledge of the political and epistemological dynamics at play between the galley and the field of contemporary art, their everyday codes allowed them to identify personally relevant themes. For instance, a 68-year-old widow stated:

Personally I liked the trolley and the walking stick because it must have took such a lot of knitting and working out. Then the rambling [a pair of walking boots] as the lady said she’d lost her husband so that brought memories of him back while she was making that.

The pieces were made by women of a similar age to the group, which provided the participants with a particular insight into the artworks. For example, this respondent had lost her husband, as had the person who had made the piece (an experience that older women share as on average men do not live as long^11^). Likewise, many respondents knitted and were impressed by the level of technical competency shown in constructing the pieces. This led to a discussion about knitting where respondents spoke about learning to make clothes at school, mentioning that it was cheaper than having to buy them when money was short. In such discussion, the artwork faded from consideration, as it stimulated recollections about place and biographies.

#### 5.1.3 Responses to the field of contemporary visual art

By asserting that art should only be defined through particular characteristics (e.g., mimesis) that are not perceived to be present in contemporary visual art, the respondents in this grouping are attempting to apply a concept of art learned through previous experience. It might be too strong an interpretation to say that they are attempting to subvert (Swartz, 1997) the field of contemporary visual art because they are unable to recognise its codes. Yet, the comments of the 73-year-old from the “live at home” scheme come close to that, when declaring what is and is not art (see Section 5.1.1). However, the discussion took place within a contemporary art gallery, and he was challenging the significance of what he observed being displayed. He recognised that, because the artworks were present in the gallery, then other people considered them as important, which prompted him to make a case for his views on what constitutes art.

However, the typical approach of this grouping to view the contemporary work as “not art” was not unchanging, suggesting a reappraisal of what constitutes art and, hence, the field of art. Indeed, there was evidence that a number of the participants learnt some of the codes of contemporary visual art unconsciously without outwardly expressing any desire to learn more about the art form and being apparently unaware of their field positions. One example, in particular, captures this nicely. After her initial visit to the BALTIC Centre for Contemporary Art in response to the *A Needle Women* (avant-garde video art), a 68-year-old female stated: “It was pleasant, but I couldn’t see no point in it personally. It was a woman standing there with her back to you. To me the cameraman's doing the work.” This work meant very little to her, as she was unable to decode the artwork. Her second visit was to the Shipley Art Gallery, where her knowledge of baking made her more appreciative of how the works had been constructed:

I thought the spoons and the baking outfit [were] really something because they looked like wooden spoons but really they weren’t, they were wired. I just thought it was very clever all the way through.

While this artwork is still being interpreted on a personal level, her ability to make sense of the art may have given her confidence and she might have unconsciously been internalising some of the rules of the field. When she visited the show by Chad McCail (*Robots run Zombies for Wealthy Parasites*, 2002—avant-garde posters representing societal control) at the Northern Gallery for Contemporary Art, she was able to discuss the artwork in terms of the intention of the artist:

They were full of life when they started, and as they got through the system they were slowly changing into black that means they were getting more downtrodden. The teachers were completely black. At the top of the school, only the tops of their heads were red. I think as an artist he was very much into *1984* the way he looks at things. Big Brother that's what I was thinking of, not Big Brother on the telly but Big Brother in the future, you know.

This demonstrated how one of the working class older participants was able to decode the art form in the final of the three visits responding more like the middle class younger group. The result of encountering a field that she was unfamiliar with was a modification of her habitus (Reay, 2004). The nature of the artwork concerned must influence the ability of a respondent in this position to achieve what she did. This response might not have been possible with art that demanded more specialist knowledge to understand it. However, this modification of her habitus changed her responses that were initially similar to the rest of the working class older grouping. According to Bourdieu (1977), reflexivity occurs at moments of mismatch between habitus and field. A process that might normally be unconscious becomes conscious. However, in this instance, the mismatch appears to have been resolved unconsciously, perhaps through the internalisation of the rules of the art form. This reformulation of the self is assumed by Marcia (2002) to be difficult for older people, which is not the case in this instance.

### 5.2 Middle class younger participants 60–65 years: the film group

All of this group, except for one, were educated to degree level, and they demonstrated a wide cultural engagement across different art forms (with occupations classed as NS-SEC 1 and 2). This is illustrated by a 62-year-old female who had been a civil servant and who, when speaking about her cultural activities, stated:

Cinemas, theatre, dance. I like modern dance. Every now and then, I get fed up with Newcastle, and I go down to London for a cultural blast. You know Newcastle's fine. I’ve lived here for thirty-odd years, but it is small so sometimes I want what only London can provide.

A 64-year-old former teacher who was also a member of this group stated:

I used to be a playwright, in fact, I still am a playwright, and I’ve just got two plays on the go at the moment. I’ve recently done a course on the Renaissance period, and that was fascinating because it was an area that I knew very little about, and so I’ve been seeking out these pictures, the Titians and the Tintorettos in the National Gallery.

The response of this group to art was one of continual discovery, and it was this context that framed their engagement with contemporary visual art as part of the research project.

#### 5.2.1 Definitions of art

Within this group, there was a general reluctance to define specific characteristics of contemporary visual art that might preference one artwork over another. The working class older groups’ propensity to compare unfavourably what they were looking at with rear-guard or consecrated avant-garde works was less evident. For some, not being immediately able to decode an artwork did not necessarily mean that it had no value.For example, as part of the baseline data collection, a 63-year-old female, who had been a social worker, stated:

If something is recognised as being a wonderful piece of art, and I don’t get anything out of it myself…I do struggle with that, but I think it's about being open you know. You were saying that people walk past and say, “What a load of rubbish;” I would never say that about anything.

This respondent professes that she struggles with art pieces that she cannot immediately decode and cannot see what others apparently see. Despite the difficulties she faces, her response is not to dismiss the art, as the group of working class older participants might have done. Openness to forms of art, which she cannot immediately decode, might be viewed as a necessary pre-condition to improving her field position. This individual has adopted a relativist stance, in terms of art, which according to Greenfield (1989, p. 171) “makes it difficult for them to pass individual judgements.” This approach was exhibited by all but one of the respondents in this group (a 64-year-old male retired engineer) who made judgements about the exhibitions at the BALTIC.

#### 5.2.2 Decoding of artworks/interpretations of art viewed

The habitus of these participants had embedded codes that allowed most of them to engage with the field of contemporary visual art in a way that many of the group of working class older participants, described above, could not. Most notably, the codes used by the majority of this group to interpret contemporary visual art incorporated some understanding of the rules of this specific field, as well as the wider cultural field with which they engaged. This meant that they did not use their personal/class histories to make sense of the artworks but, instead, attempted to interpret them in terms of the cultural capital that they perceived was relevant. This led to in-depth discussions of the various art pieces they came into contact with. The response to Jenny Holzer's exhibition (avant-garde video art) held at the BALTIC Centre for Contemporary Art by a 63-year-old male retired teacher is indicative. He stated:

I think the more that you spent time with the installation, the more you got from it. She said there was five hours worth of text, that's a lot of text but at the same time it had a kind of mesmeric and hypnotic quality about it. You couldn’t take your eyes off it.

This approach was representative among the other five participants in this group, representing a willingness to engage with the field of contemporary art on its own perceived terms.

#### 5.2.3 Responses to the field of contemporary visual art

A number of the participants consciously attempted to improve their field positions. Their recognition of the field, and a sense of the sort of cultural capital that was valued within it, encouraged them to learn more about the art form. A number of members, such as a 65-year-old retired teacher, normally carried out research on artists and exhibitions before visiting. This individual referred to the visits associated with the research project as a course, stating:

Well I think partly because of this course that we are doing now, I’m just trying to educate myself in seeing as much contemporary art as I can so I have a better perspective on what we’re going to be looking at.

The visits were viewed by some participants as a way of exploring and developing their understanding of the art form, seeing the visits as an educational experience. During discussion, it was evident that some of the younger participants were conscious that their knowledge was partial. This 62-year-old female, who had been a civil servant, commented:

I don’t feel at all confident about choosing what to go and see or experiencing what I’m experiencing and talking about that because I don’t know anything about it. So this is a dip-a-toe-in-the-water kind of experience for me, and it's relaxed and social and there's a nice lunch, which is great, and yes, it makes me feel more confident about going to things like this.

The field strategies of those identified under this theme were a combination of conservation and succession (Swartz, 1997, p. 125). While some spoke confidently about the pieces on display, they were conscious of their limited knowledge. They responded to this realisation by taking steps to increase their knowledge. It is theorised that their engagement with other cultural forms—such as theatre and film—and their take-up of educational opportunities—such as adult education classes—transforms their habitus, giving them the tools to deal with this situation incrementally. The investments that are made in cultural capital ultimately result in a stronger field position, not in the sense that an individual becomes an actor in the field such as a curator or artist (although this may happen), but rather in the sense that they negotiate the field more comfortably and with greater sense of personal gain. While there is a lack of confidence amongst some in this group, these feelings are not expressed in terms of exclusion, as the codes they apply are able to recognise and engage with the field to a particular level. Their habitus might be labelled as “reflexive,” as described by Sweetman (2003) and Bottero (2010). It contained within it the ability to adjust—to decipher codes associated with art forms that they were not immediately familiar, perhaps being able to recognise commonalities.

The middle class younger group and the working class older grouping demonstrated very different positions within, and dispositions towards, the field. The differences in respondents’ habitus can be attributed to early socialisation and their life course experience, which influence responses. For the older and younger groups discussed in these first two sections, the differences in cultural capital probably originate in their experience of education, which had differed considerably.

### 5.3 Participants demonstrating class mobility over the life course: the writers’ group

Evidence of class mobility was seen in a number of the groups in this study. However, it was most clearly observed affecting the responses of the writers’ group from Sunderland. This group had one member who was 64, and the others were between 74 and 87 years-old, and all being female. They described themselves as working class and originally had occupations that could be classified as such, secretarial work, for example. However, some had returned to formal education later in life and had taken middle class occupations. A 74-year-old member of the group stated “I’ve always considered myself working class but I was a probation officer for 20 years.” She had also taken a module on history of art as part of her degree.

#### 5.3.1 Definitions of art

This group had a preference for particular forms of rear-guard or consecrated art in general, as illustrated by the 74-year-old member of the group mentioned immediately above, who stated:

Well, I did history of art for my degree so I always wanted to see the things I’d studied, and I eventually got to Italy but it was my own present to my-self on my sixtieth birthday. I’d done my degree twenty year before that so I waited twenty years to see the things I wanted to see. So I went to Florence, and I saw Brunelleschi's dome, the Baptistery and the bronze doors, which I loved.

An 81-year-old who had worked in shops and factories was open to the possibility that avant-garde art has value, but she was looking for the characteristics that could be assimilated with rear-guard or consecrated art forms with which she was familiar. She states: “I look for the beauty in art, but modern art; if I can see something I can try and understand, but there's so much I don’t understand in modern art.” However, other members rejected some famous avant-garde art pieces of which they were aware. A 74-year-who returned to formal education in middle age stated about *My Bed* (1998) by Tracey Emin, “The unmade bed is just out the window you know.^12^” This understanding of art reflects the participant's working class origins and mirrors, to an extent, the responses of the working class older group described above. However, members of this group have greater stocks of cultural capital associated with particular rear-guard or consecrated art, particularly the member who had studied art history.

#### 5.3.2 Decoding of artworks/interpretations of art viewed

The codes they were using to interpret the artworks did not enable the same level of engagement as those used by the film group (Section 5.2), and they did not articulate a conscious desire to learn about the art form and the intentions of the artist. However, they were mostly able to discuss the artworks that they viewed rather than rejecting them—thereby departing from the working class groups who lacked such upward mobility.

When they visited the BALTIC Centre for Contemporary Art they discussed Kimsooja's *A Needle Woman*—a video installation showing the artist from behind standing still in the human traffic of busy streets in different highly populated cities^13^—they entered into a detailed discussion of the artworks. This consisted of how the people responded to the camera and the clothes that were being worn. The 81-year-old stated:

Yes there was differences but the others [i.e., the passers-by in the videos] were quite relaxed and just getting on with life, and what struck me was they were all dressed the same all over the world. You can go to Nigeria and they don’t dress in Nigerian clothes, long robes or anything. Everybody's in Western dress, bright colours.

Typically, while respondents were unable to decode the artworks in terms of the field of contemporary art, the codes they applied enabled them to make personal sense of what they viewed—but not by employing the particular, everyday codes that the working class groupings did.

One code this group employed drew upon their experiences of class divisions. In response to their visit to the Northern Gallery for Contemporary Art to see *Rank* (a combination of rear-guard, consecrated avant-garde and avant-garde artworks revolving around a central exploration of representations of class difference and inequalities), they used the theme of the visit as the basis for a wide-ranging discussion about their own experiences and views. For example, they mentioned the importance of education in changing society, with the former probation officer stating:

When I went into further education, I was in my late thirties and I did sociology. I thought I was quite happy with my lot [laughs] till I did sociology at college and then I realised that I shouldn’t be happy with my lot because I was hearing about how two percent of the people own ninety percent of the wealth; then I began to question it.

The inequality in access to healthcare because of cost was mentioned a number of times. The 87-year-old who attended university at the age of 62, stated:

Oh, it cost a fortune you know. I remember my sister….had to get a few teeth out and the dentist came to the house and we were finding teeth all over the place because he just stood there and he was tossing the teeth out as he pulled them [laughs], and we were finding teeth all over because she got them all out. Oh dear, what a carry on but we paid for everything.

The discussion did not engage with the art itself—despite participants having the cultural capital associated with certain types of rear-guard and consecrated avant-garde works. The sort of rear-guard and consecrated avant-garde art displayed did not appear to be accessible to them. Yet, their everyday codes did draw upon their mobility experiences when attempting to make sense of such art.

#### 5.3.3 Responses to the field of contemporary visual art

Because members of this group appear to have the cultural capital associated with a limited range of rear-guard and consecrated avant-garde works and not for avant-garde art, it is not possible to observe a particular field strategy being adopted, as defined by Swartz (1997). Their responses appear to be influenced by their working class origins and then modified through later class mobility. An important aspect of this appears to be the taking up of educational opportunities—for example, attending university in middle age. This has been made possible by large-scale educational reforms in the post-war period (Goldthorpe et al., 1987), which have strongly influenced the lives and individual experience of this group. As Scherger and Savage (2010, p. 420) state, “higher education boosts the odds of being upwardly mobile.” This has provided some these respondents with greater stocks of cultural capital associated with particular rear-guard and consecrated avant-garde works. Friedman (2012), drawing from an empirical study of British comedy tastes, found that certain tastes are established in childhood and then added to as cultural capital grows. In this case, taste associated with visual art does not appear to have changed. However, their stocks of cultural capital associated with that taste have increased, while other art forms, such as creative writing have been added to their repertoire.

### 5.4 Mixed class/age group: advocacy organisation for older people

Within this group, three participants were aged 63 and 64 while 6 were aged between 79 and 83. Included in the older group were two local government officers and a woodwork teacher (NS-SEC groups 3 and 4) and in the younger group a university researcher and a person who worked in private industry (NS-SEC groups 1 and 2).

#### 5.4.1 Definitions of art

There was a range of views expressed about the significance of contemporary visual art within this group. In the baseline focus group, a 79-year-old female retired local government officer stated about Sam Taylor-Wood's *Ascension* (2003)—which she had seen at the BALTIC Centre for Contemporary Art—“There was somebody tap dancing on a board on top of somebody lying down. It wasn’t art in my mind.” This resonates with the older working class individuals in the opening section. However, the middle class younger members of the group often had a different opinion, as is illustrated by a 64-year-old female who had worked in private industry. She enjoyed walking with a friend and remarked on the public art that her friend often came across.

She's not very keen on contemporary art, so we have a few discussions over things like that. It's nice if there is sometimes a little plaque [i.e., label] or something to give you some idea, particularly if you’ve really got no idea what it is just to give you some idea what the artist did have in mind. Whether you agree with them or not that's a matter of opinion, but I still think it's nice to find out what they thought they were doing.

While this respondent lacked the cultural capital to fully engage with what she had seen, she did not dismiss it out of hand, and she was interested in finding out more about the artist's intention when producing the piece. Her definition of art encompassed more avant-garde works that she was willing to explore further. This is a similar response to that seen in the middle class younger group described above.

#### 5.4.2 Decoding of artworks/interpretations of art viewed

The ability of the respondents to decode the work, and so produce interpretations of what they saw, was divided along the lines indicated above—with some turning to everyday codes and others turning to their stock of cultural capital. In a focus group that took place at the BALTIC Centre for Contemporary Art, for example, an 83-year-old retired civil servant made a point of discussing a painting he had studied in an art history course, Jan van Eyck's 1434 *Arnolfini Portrait* in the National Gallery in London. He states:

If you look at the mirror on the wall, it's got the little inscription and it says, “van Eyck was here.” If you look carefully at the mirror, there's the image of a young man behind the door who is actually the lover of van Eyck, he's a skinny little man dressed in a brown cloak trimmed with ermine.^14^

This discussion of consecrated rear-guard work in a contemporary art gallery represents an attempt to move the conversation to, what was for him, familiar ground. It also appeared important for this participant to demonstrate his knowledge, possibly in a play for group leadership.

This approach was not mirrored by two of the three younger members (the third was less confident and articulate), who were analytical in how they discussed the artwork. As an example, the 63-year-old retired private industry worker gave a sophisticated and self-reflexive response to *Heliocentric* (avant-garde video art):

I just felt so small and insignificant, particularly when you’ve got the one where you saw the sun so close to but you could make it [out] as still a sphere and I just thought that was so remarkable.

#### 5.4.3 Responses to the field of contemporary visual art

Those with the cultural capital to engage with the field had strategies similar to the middle class younger group discussed above, that being a combination of conservation and succession (Swartz, 1997). Those without it could observe that avant-garde art existed, but were unable to engage with it on its own terms. However, over the course of the three visits to galleries, the discussion became increasingly dominated by the two younger members, and their approach was adopted by the third younger, as well as the older, members. For example, the 83-year-old retired civil servant, mentioned above, became more reflective by the end of the visits. He stated at the last focus group that took place at Belsay Hall and Gardens about *Extraordinary Measures*, an exhibition on the theme of scale.

If the exhibition had been moved to a more modern environment would it have changed the exhibition itself? If you looked at it in the BALTIC for example would it have had the same impression?

This represents a willingness to engage with the art and a change from his comments above, when he attempted to move the discussion to consecrated rear-guard art that he was more familiar with.

It is difficult to be confident as to why the views of the two younger members of the group dominated the discussions as opposed to the older ones. However, their habitus was characterised by greater reflexivity, which enabled them to comment articulately and persuasively on what they had seen. This may have been the product of greater educational attainment that was generally a characteristic of the middle class younger participants. It may also relate to members becoming more aware of the field during the visits and the two younger members being seen to be more able to decode it. The social dynamics of this situation reflect the results of Hekkert and Wieringen (1998, p. 281) whose study found that when discussing the quality of artworks “latter judgements often reflected the original evaluation of only one of the group members.” In event, these findings resonate with Bottero's (2010) point regarding the impact of group dynamics on habitus.

### 5.5 Ageing effects seen across different groups

The sections above considered the responses to contemporary visual art of groups or groupings of participants with certain characteristics. However, it is possible to identify a response to artworks that cuts across the groups. Ageing effects are defined by Scherger (2009) as being physical, psychosocial and social. While not often discussed, they were present in some responses of the participants. Some of them across the different class/age groups used the experience of the visits to respond to real or perceived age associated deficits that might interfere with their sense of self. Angus et al. (2005, p. 184) state that, “ill bodies may gradually or abruptly lose the capacity to enact deeply inculcated dispositions associated with a particular social position, especially those associated with class or gender.” For example, a 65-year-old member of the film group, who was previously a teacher, saw cultural engagement as a response to feeling that he was running out of time. He states:

You can go on courses like this and I love the freedom of being able to learn, but the desire to learn is probably more with me now than it was when I was younger, I think maybe because time's running out. There is going to come a time when Alzheimer's sets in or you get the Iris Murdoch syndrome^15^ when you start to forget things.

Members of the writers’ group thought it was important to keep mentally active in order to maintain their mental faculties and visiting museums and galleries was a way of achieving this. An 87-year-old stated:

I’m the oldest here being in my eighties. I feel that these things have kept me going because I would just be sitting at home sleeping or doing nothing if I hadn’t this education, all these museums and places we go to, they are wonderful.

The need to respond to possible future deficits might be seen as an example of Ekerdt's (1986, p. 26) “busy ethic” with its “emphasis on activity, exercise, travel, eating out, self-maintenance and self care.”

An example of how engagement with the content of particular artworks helped an individual to respond to the effects of ageing is in the following response to the *Knitted Lives* exhibition at the Shipley Art Gallery. She is a 79-year-old wheelchair user who is a member of the sheltered accommodation group. She states:

I think I’d start taking it [knitting] up again…But I know I haven’t got the use in this hand that I did since I had the stroke—but I wouldn’t mind trying it. And then my memory's not what it used to be.

For this respondent, being able to knit provided a link to her younger self when she had made clothes for herself and her family. The physical aspects of ageing had interrupted her construction of self. Despite thinking that she would not be able to knit, she was able to use the needles and wool provided as part of the exhibition. It was her confidence, more than her motor skills that had declined. The above can be viewed as attempts to maintain a particular ingrained habitus that might be threatened by age related changes.

## 6 Discussion and conclusion

The above analysis shows that, for the field of contemporary visual art, both habitus and cultural capital shape how older adults respond to works associated with that field—with those relatively rich in cultural capital, and those with a habitus that extends to non-representational art, displaying more openness, if not comfort, when confronting and “decoding” those contemporary works. The respondents’ origins—including their class and generation, and sometimes gender—left an imprint on their cultural capital and habitus, as Bourdieu would expect. However, cultural capital and habitus are not immutable (see Reay, 2004). Some respondents, for example, would add to their stock of cultural capital by returning to school later in life, and others would develop new ways of classifying and appreciating art—with the latter occurring in the wake of class mobility and group dynamics (see Bottero, 2010). The qualitative nature of this study means that conclusions about the relative importance of such things as habitus or mobility, in particular circumstances, are not possible to make—but it is clear that they do matter for these respondents.

A division between an older grouping comprising those from the sheltered accommodation and the “live at home” scheme and a younger group comprising those from the film group could be observed. These respective groups were largely homogeneous in terms of class and age represented particular profiles in each. While broad similarities within class/age groupings can be identified, as Scherger and Savage (2010, p. 424) state, “classes are not monolithic; they do not show completely consistent and uniform patters of cultural participation and socialisation.” Those in the mainly working older class grouping (sheltered accommodation and the “live at home” scheme) either rejected the artworks or interpreted them using aspects of their habitus that allowed them to make sense, in their own terms, of what they were observing. Meanings associated with art pieces that were nothing to do with the field of art have (e.g., whether a painting matched the furniture) have been identified by Halle (1993)—results that are replicated in this study when these respondents relied on codes of everyday life rather than those aligned with art. It can be theorised that such meanings are not serendipitous but associated with identity processes described as “maintenance” and “revision,” as understood by Kroger (2002), Kroger and Adair (2008), Marcia (2002), and in terms of art interpretation, Newman et al. (2013). In this case, by making links to their pasts, participants were able to maintain a sense of continuity over time, which is important for the wellbeing of older people. Note that those in the working class older grouping would not have attended the venues and shows without the occasion of the research project. The sheltered accommodation group were observably tense during their first visit (to the BALTIC Centre for Contemporary Art), which was evidenced by raised voices indicating that they felt out of place; but this was not seen in subsequent visits as their knowledge and confidence grew. Their enjoyment increased markedly if they could use their existing codes of everyday life to make sense of what they saw.

Those in the middle class younger (film group) category were more able to recognise the field of contemporary visual art. They attempted to engage with it more on its own terms, often being conscious of and attempting to improve their field position. Their embedded codes—often derived from an associated art form such as film—appeared, to an extent, to be transferable in providing interpretative tools to the genre of contemporary visual art. The ability to transfer codes from one art form to another and general attempts to improve their field position requires a degree of reflexivity that does not appear to be available to all of the respondents in equal measure and, according to Wee and Brooks (2010), can been seen itself as a form of cultural capital. This characteristic is mainly concentrated in responses of the middle class younger participants and is possibly associated with attempts, within constraints, to “actively fashion” (Wee and Brooks, 2010, p. 47) their identities. However, for some, in the working class older participant groups, reflexivity, as cultural capital, appeared to be acquired unconsciously over the three visits. This is an area that would benefit from further research.

Class mobility also had an impact upon attitudes towards the field and positions with it. This was observable across many of the groups, but particularly in the writers’ group. Three members had taken advantage of educational opportunities with one then taking a professional job considered as middle class. They used the artworks for reminiscence and also attempted to understand their meaning in terms of the field, strategies used by the working class older and middle class younger groups. Van Eijck (1999, p. 312) states that, “the cultural behaviour of upwardly mobile individuals is predicted on the significance of the mobility itself.” Cultural activities for this group were not a product of mobility but, instead, were embedded within mobility. They were proud of the status that the activities of the group gave them and mentioned this in the baseline interviews. Conditions that encouraged mobility were particular generational experiences, such as access to education.

The social dynamics within the group appear to affect changes in cultural capital associated with contemporary visual art over the visits. This was most visible in the group comprising the advocacy organisation for older people being mixed in terms of class and age. However, it was also present in the other groups—such as the middle class younger group, within which there was a disparity between some members’ knowledge about contemporary visual art. It is possible that the more heterogeneous the group, then the greater the potential for the relevant stocks of cultural capital (in its embodied form) amongst some members to be increased. Similarly, habitus within groups was becoming co-ordinated (Bottero, 2010; King, 2000) in terms of their response to artworks. It is also possible that social capital, in terms of the relationships between group members, is being translated into cultural capital (in relation to ways of approaching contemporary visual art) (Bourdieu, 1997) as the visits progressed. The significance of social capital for older people has been explored by Gray (2009) and Nuqvist et al. (2006), while how cultural tastes shape personal networks is analysed by Lizardo (2006). The latter describes the conversion of “cultural into social capital” and concludes, “different forms of cultural consumption can lead to different types of ego-network structure and composition” (Lizardo, 2006, p. 803).

Some of the participants used the experience of engaging with museums and galleries to respond to real or anticipated age-associated deficits. With one instance of a participant making links to her younger self in response to the *Knitted Lives* exhibition and re-establishing aspects of her identity that had been lost.

The above analysis presents a picture of cultural consumption by older people that complements that provided by the quantitative studies described earlier in this article (Keaney and Oskala, 2007; Scherger, 2009). While broad patterns can be identified, the complexity of how some differences influence consumption is illustrated. For example, a number of large-scale studies contend that formal educational attainment is a marker for engagement with culture (Bennett et al., 2009; Chan and Goldthorpe, 2007; DiMaggio and Mukhtar, 2004). This can be seen with the film group, who were, apart from one member, educated to degree level and demonstrating greater engagement than the sheltered accommodation and “live at home” scheme groups who left school at age 14 or 15. However, those with less formal education still demonstrated a liking for art with a preference for rear-guard and consecrated avant-garde work. The nature of their education is also complex, with members of the writers’ group leaving school at 14 or 15 with some returning to formal education later in life. All had taken advantage of adult education classes during their lives. Their formal educational attainment could not be simply correlated with engagement with various art forms. Their learning was often informal and, therefore, could not easily be captured.

UK Arts policy (Arts Council England, 2010) privileges understandings of contemporary visual art that relate to the knowledge associated with the field, as would be predicted. However, if galleries wish to widen their audiences and overcome barriers to engagement, it is important for them to recognise that the meanings that many associate with artworks may have little to do with the field. This may be overcome by providing themes that can be interpreted using everyday codes rather than field-specific ones. Interpretation, either through labels or guided tours, facilitates this process. It is evident that, in some instances, exposure to contemporary visual art allows the unconscious learning of its codes, which can facilitate access to the field.

None of the participants was from black and minority ethnic groups. When asked they declined to become involved in the research. When asked why, a group of women of Pakistani origin from Gateshead replied that the exhibitions were of no cultural relevance to them. However, if crafts from their home country were being shown they would have attended. This is an area that requires further research and has been so far been addressed by Banks (2010), Bennett et al. (2009), Grams (2010), and Trienekens (2002).

As for the present article, it is hoped that these insights will help to inform the development of educational and interpretive resources in art galleries, as well as to improve our understanding of the actual and potential importance of cultural content (e.g., contemporary art) in older people's lives.

## Footnotes

1 http://www.newdynamics.group.shef.ac.uk/.

2 Dunleavy and Hood (1994, p. 9) describe the New Public Management as a “way of reorganising public sector bodies to bring their management, reporting and accounting approaches closer to business methods”.

3 http://www.open.ac.uk/socialsciences/cultural-capital-and-social-exclusion/project-summary.php.

4 http://www.culture.gov.uk/what_we_do/research_and_statistics/4828.aspx. This quantitative survey has been running continuously since 2005 and surveys about 29,000 people (aged 16 and above).

5 This publication was produced by the UK Office for National Statistics, and it provides categories that are accepted in the UK http://www.statistics.gov.uk/hub/index.html.

6 Guidance on capacity was provided by specialists in Newcastle University's Institute for Ageing and Health. All participants gave informed consent and signed a form agreeing to take part.

7 This is software designed to help manage qualitative data (http://www.qsrinternational.com/).

8 http://www.ons.gov.uk/ons/guide-method/classifications/current-standard-classifications/soc2010/soc2010-volume-3-ns-sec--rebased-on-soc2010--user-manual/index.html.

9 “O” levels and “A” levels are examinations taken by children in British schools at ages of 16 and 18 respectively—although “O” levels were replaced by the General Certificate of Secondary Education (GCSE) in 1988 in England, Wales and Northern Ireland.

10 This effect relates to changes in the minimum age for leaving school. The 1944 Education Act (enforced in 1947) increased the leaving age from 14 to 15—therefore, in 2009 (when we started the project), those 77 and younger could have left school at 15, while those older could have left at 14.

11 http://www.ons.gov.uk/ons/rel/mortality-ageing/focus-on-older-people/older-people-s-day-2010/index.html.

12 The respondent feels that this piece has no value: http://www.saatchi-gallery.co.uk/artists/artpages/tracey_emin_my_bed.htm.

13 http://www.balticmill.com/whatsOn/past/ExhibitionDetail.php?exhibID=135.

14 To the authors’ knowledge, there is no published support for this interpretation.

15 Iris Murdoch (1919–1999) was an author and academic who suffered from Alzheimer's disease.
