# Supplementary material for: How cultural capital, habitus and class influence the responses of older adults to the field of contemporary visual art
Source: Poetics (Amst). 2013 Oct;41(5):456–80. doi: 10.1016/j.poetic.2013.07.001 (PMC3990446; doi:10.1016/j.poetic.2013.07.001)
Supplement: Supplementary file 1 [file mmc1.pdf]

POETICS *Journal of Empirical Research on Culture, the Media and the Arts*

ONLINE SUPPLEMENT TO ARTICLE IN POETICS VOL. 41 (2013) no. 5

How Cultural Capital, Habitus and Class Influence the Responses of Older Adults to the Field of Contemporary Art

Andrew Newman, Anna Goulding, and Christopher Whitehead

University of Newcastle upon Tyne

Appendix A

The Venues and Shows Visited by the Five Groups of Older Adults

Northern Gallery for Contemporary Art, Sunderland, Tyne and Wear

Opened in 1995 as part of the Sunderland City Library, it displays mainly avant-garde art—although *Rank* (see below) also displayed consecrated avant-garde and rear-guard forms. Its location makes it less intimidating to those who would not normally visit art galleries.

- *Rank, picturing the social order 1516-2009*[1] explored inequality in society.
- *Semiconductor*[2] are Brighton based artists Ruth Jarman and Joe Gerhardt who explore scientific knowledge through video.
- *Heliocentric* is a three-screen installation that uses time-lapse photography and astronomical tracking to plot the sun's trajectory across a series of landscapes.
- *Systematic* by Chad McCail. Explores how "society produces and fails to produce 'normal' individuals who accept its rules."[3]

---

[1] http://www.ngca.co.uk/home/default.asp?id=147

[2] http://www.artrabbit.com/all/events/event/17805/semiconductor_heliocentric

<u>BALTIC Centre for Contemporary Art, Gateshead, Tyne and Wear</u>

Housed in a remodelled, disused flour mill situated on the banks of the River Tyne, it is a "white cube" gallery[4] and displays avant-garde art.

- *Parrworld*[5] was produced by Martin Parr and consisted of a collection of photographs and assorted objects documenting historical and political moments and secondly an exhibition entitled *Luxury*, showing the different ways in which people display their wealth.
- *A Needle Woman*[6] by the Korean artist Kimsooja. Consisting of eight simultaneous videos, it documents the artist as she stands motionless in the crowded streets of Lagos, Mexico City, Cairo, New York, Delhi, Tokyo, Shanghai, and London.
- Jenny Holzer[7] used electronic text to explore themes such as authorship, power, hope, despair, need and longing.
- Cornelia Parker[8] transforms familiar everyday objects, interrogating the meanings society gives to them.
- Anselm Kiefer[9] is interested in myth, history, theology, philosophy and literature, and his work consists of painting, sculpture and installation.

---

[3] http://www.ngca.co.uk/home/default.asp?id=160&prnt=18

[4] O'Doherty, B., 1999. Inside the White Cube: The Ideology of the Gallery Space. University of California Press, Berkeley, CA.

[5] http://www.balticmill.com/whatsOn/future/ExhibitionDetail.php?exhibID=130

[6] http://www.balticmill.com/whatsOn/past/ExhibitionDetail.php?exhibID=135

[7] http://www.balticmill.com/whatsOn/past/ExhibitionDetail.php?exhibID=136

[8] http://www.balticmill.com/whatsOn/present/ExhibitionDetail.php?exhibID=141

BALTIC is highly prestigious, attracting international artists, and hosting the Turner Prize[10] in 2011. It has an international position in the field of contemporary visual art, and it was intimidating for some of the respondents who had not previously visited.

Shipley Art Gallery, Gateshead, Tyne and Wear

Opened in 1917 and is now managed as part of Tyne and Wear Archives and Museums.

- *Knitted Lives,*[11] this consists of a range of knitted everyday objects, such as a shopping trolley—produced by 32 older women from the region (avant-garde)

The gallery specialises in contemporary craft showing a combination of consecrated avant-garde and avant-garde works, and it has a national reputation in craft. The gallery has an established relationship with its local community, and it did not intimidate those who visited.

Belsay Hall, Castle and Gardens, Northumberland

Owned and managed by English Heritage, the hall, castle and gardens have been used for art installations each summer, since 2004.

- *Extraordinary Measures* is a collection of artworks on the theme of scale (avant-garde).

The venue has built up a local reputation for its summer art installations.

---

[9] http://balticmill.com/whatsOn/past/ExhibitionDetail.php?exhibID=145

[10] http://www.tate.org.uk/whats-on/tate-britain/exhibition/turner-prize-2012

[11] http://news.bbc.co.uk/1/hi/england/8021534.stm

Great North Museum: Hancock, Newcastle upon Tyne, Tyne and Wear

Managed by Tyne and Wear Archives and Museums, it was chosen for a visit by the writers' group from Sunderland. This museum shows mixed collections—for example, world cultures, natural history, archaeology and geology.
